# Micro- and Macroalgae in Meat Products

**DOI:** 10.3390/foods13060826

**Published:** 2024-03-07

**Authors:** Caba Siladji, Vesna Djordjevic, Jelena Babic Milijasevic, Volker Heinz, Nino Terjung, Weizheng Sun, Igor Tomasevic

**Affiliations:** 1 Institute of Meat Hygiene and Technology, Kaćanskog 13, 11000 Belgrade, Serbia; vesna.djordjevic@inmes.rs (V.D.); jelena.babic@inmes.rs (J.B.M.); 2 DIL German Institute of Food Technologies, Prof.-v.-Klitzing-Str. 7, 49610 Quakenbrueck, Germany; v.heinz@dil-ev.de (V.H.); n.terjung@dil-tec.de (N.T.); 3 School of Food Science and Engineering, South China University of Technology, Guangzhou 510641, China; fewzhsun@scut.edu.cn; 4 Faculty of Agriculture, University of Belgrade, Nemanjina 6, 11080 Belgrade, Serbia

**Keywords:** *Chlorella*, *Spirulina*, Wakame, Sea spaghetti, Sea tangle, Nori, sausages, frankfurters, Patties

## Abstract

Technology in the meat industry is advancing to create healthier and more sustainable food. Incorporating micro- and macroalgae into meat products presents an exciting possibility for the meat sector to develop functional food, given that they serve as excellent natural sources of nutrients and bioactive compounds. This review aims to systematically outline the impact of incorporating whole algae and their extracts into various meat products, examining their effects on quality, physicochemical and functional properties, sensory characteristics, and potential for enhancing shelf life. Adding algae to meat products generally increased pH values, with variations influenced by concentration, type, initial pH, and storage time. The protein content was mainly unaffected, except for Nori and *Chlorella*. Algae contributed to lower moisture and higher ash content due to dietary fiber. While including algae improved water-holding capacity and decreased cooking loss, it often led to increased hardness and chewiness. Algae and their extracts influenced color attributes, with variations based on the algae type. Sensory properties were distinctively affected, generally reducing overall acceptability, although Sea tangle at concentrations of 1–3% showed acceptable scores. *Chlorella* and Sea tangle positively impacted microbiology during refrigerated storage, while algae and their extracts demonstrated strong antioxidant activity.

## 1. Introduction

Due to its high nutritional content and delicious flavor and texture, meat has always had a special place in the human diet. It is an excellent source of dietary protein and has a high biological value. In addition to being a significant provider of proteins, vitamins, and minerals, meat also contains fat, particularly cholesterol, triacylglycerol, saturated fatty acids (SFA), and phospholipids. However, people more and more frequently have a negative perception of meat products as a high-fat, high-salt-containing food [1].

The development of “healthier food products” has witnessed remarkable growth. These must possess one of the following qualities: altered composition and/or processing conditions to prevent or limit the presence of certain potentially harmful compounds and/or the ability to incorporate desirable substances, either naturally occurring or added, that offer further health benefits. The concept of “healthier” products includes what is referred to as “functional foods” [2]. Technology has continued to evolve in the meat industry to produce healthier and more functional meat products to address public awareness of the relationship between nutrition and health. Meat products can be enhanced and reformulated with bioactive components while partially reducing fat and salt [3].

Depending on their size and appearance, algae are typically classified into microalgae and macroalgae. Microalgae, as the name suggests, are microscopic photosynthetic organisms, mostly unicellular. Macroalgae, on the other hand, consist of multiple cells that combine to form structures similar to the roots, stems, and leaves of higher plants [4].

Algae represent innovative foods that hold promise in meeting the macro- and micronutrient needs of the growing world population [5,6]. Since algae are an important source of biologically active compounds, they can be utilized in producing functional foods. Incorporating their natural extracts improves the quality of foods, reduces the reliance on chemical preservatives, and provides several health benefits. Therefore, using algae as a functional component can help solve several problems in meat products [7].

Algae serve as an excellent source of proteins, lipids, carbohydrates, vitamins, and macro- and microelements [8,9,10]. Numerous substances produced by the metabolism of microalgae and macroalgae have a range of beneficial health effects, including antioxidant, anti-inflammatory, anti-cancer, and antibacterial activities [11,12]. Primary phytochemicals attributed to these valuable qualities are phenolics, carotenoids, pigments, phlorotannins, and sulfated polysaccharides [11]. Apart from their important roles as natural preservatives and antioxidants, the addition of algae or their isolated components (extracts) into meat products can be an efficient way for consumers to increase the intake of bioactive substances with health benefits in their diet [3,11].

The extraction of bioactive compounds from algae excludes impurities or unwanted byproducts, which could pose challenges when integrating whole algae into foods [13].

Microalgae, such as *Chlorella* and *Spirulina,* contain high amounts of protein (up to 70% on a dry weight basis), while the protein content in various macroalgae species ranges from 10% (*Fucus vesiculosus*) to 47% (*Porphyra tenera*) [13]. Predominantly used algae ingredients are carrageenan, agar, and alginates, as these polysaccharides are used in a variety of food products as thickening, gelling, stabilizing, and packaging agents [14,15]. Algal polysaccharides are commonly used in the food industry because they are easy to extract and provide several health advantages. Green, brown, and red seaweed species (e.g., *Undaria pinnatifida, Palmaria palmata, Porphyra umbilicalis)* contain β-carotene, lutein, neoxanthin, fucoxanthin, and zeaxanthin [16,17].

This review aims to present a systematic overview of the influence of micro- and macroalgae addition in different types of meat products on their quality, physicochemical and functional properties, sensory characteristics, and potential for shelf-life improvement.

## 2. Materials and Methods

To select articles that met the criteria defined by the authors, the Preferred Reporting Item for Systematic Reviews and Meta-analysis (PRISMA) guidelines were adopted. This review includes studies summarizing the outcomes of incorporation of micro- and macroalgae, along with their extracts, into pork, beef, chicken, and turkey meat products. The primary variables used to compare products with and without algae were physicochemical and functional properties, shelf-life extension potential, and sensory properties.

The literature search was conducted between 1 November 2022 and 1 June 2023 using Scopus and Web of Science. Inclusion criteria encompassed articles in English with full-text accessibility published within the 2008–2023 period, excluding reviews. Studies exploring how adding algae and their extracts to animal feed affects meat quality were also disregarded. Only studies focusing on using seaweed, microalgae, and algal extracts in meat products were considered. Keywords used were as follows: (microalgae OR seaweed AND extracts) AND (meat OR sausage OR burger) OR (chicken/pork/beef).

**Figure 1:**
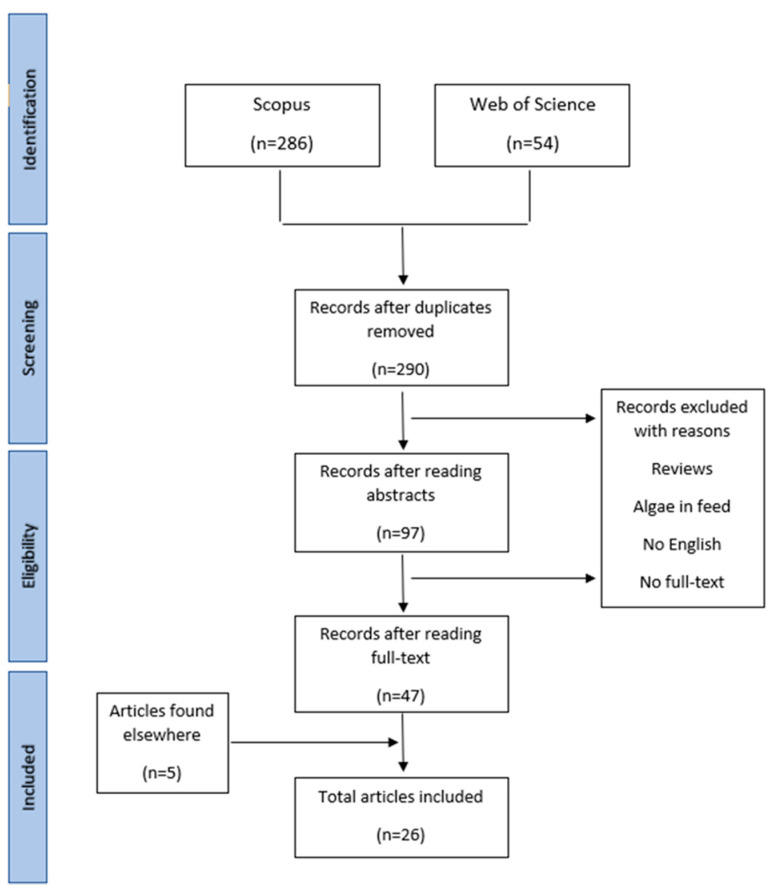
Search strategy and article selection process.

The initial search yielded three hundred forty (340) publications, and after eliminating duplicate papers that appeared in both databases, 290 studies remained. Subsequently, the abstracts of the remaining articles were reviewed, selecting those aligned with the study’s topic. Upon downloading and thoroughly examining full papers to confirm compliance with the inclusion criteria, 47 articles remained. In addition, the reference lists of the selected papers were reviewed to identify potentially relevant publications. Following a comprehensive assessment of complete texts, 26 publications were identified as eligible for this systematic review. Figure 1 illustrates the selection process flowchart.

## 3. Results and Discussion

### 3.1. pH

The pH of algae can vary from slightly acidic to slightly alkaline depending on the content of polysaccharides, minerals, polyphenols, and flavonoids [18,19]. As a result, they could affect the pH of the meat product when added to it. However, there seems to be no certainty in which direction the addition of algae affects the pH of meat products (Table 1). On the one hand, when mechanically deboned chicken meat sausages were prepared with the addition of *Kappaphycus alvarezii* macroalgae [20], a small but significant increase in pH values (0.1 max) was observed. Widati et al. [19] had a similar conclusion after incorporating 7.5% *Eucheuma cottonii* flour in Indonesian-style beef meatballs. Again, pH values significantly increased (0.13) in samples with macroalgae compared to the control samples. With the 2% addition of *Undaria pinnatifida* (Wakame), a small but significant increase in pH values (0.10) in salt-reduced frankfurters was observed [18]. Cofrades et al. [21] reported an even smaller rise in pH (+0.03) after adding the same macroalgae in a slightly higher concentration of 2.5% to low-salt gel/emulsion meat systems. On the other hand, 2.5% of *Himanthalia elongata* (Sea spaghetti) had no significant impact on pH values, while adding *Porphyra umbilicalis* (Nori) resulted in significantly lower pH values by 0.04. When *Laminaria japonica* (Sea tangle) powder was added to patties [22] and breakfast sausages [23] in a concentration of 5% and 4%, respectively, the pH levels dropped by 0.10 (*p* < 0.05). In pork liver pâtés, the pH values were unaffected by the addition of *Spirulina* at a dosage of 2.5% [24].

Where algae extracts are concerned, the addition of 0.5% polysaccharide extract of *Spirulina* to Chinese-style sausages significantly lowered the pH by less than 0.1 during 24 days of cold storage [29], while a tenfold increase in the amount of the extract in the same matrix lowered the pH by 0.24 during 18 days of storage [30]. Triki et al. [31] compared the effects of sodium chloride and a commercial replacer based on seaweed extracts (AlgySalt^®^) in fresh and cooked sausages. Contrary to NaCl, AlgySalt^®^ led to a significant decrease by 0.3 after 15 days of storage of fresh samples, while in cooked products, AlgySalt^®^ appeared to have no impact on the pH of the products. Neither the addition of laminarin and fucoidan from *Laminaria digitata* (0.5%) nor the mixture of extracts of *Ulva lactuca* and *Ulva rigida* (0.1%) had a significant effect on the pH of fresh pork patties [32,33]. The same was observed when polysaccharide concentrates (0.12%) from the green algae *Chaetomorpha linum* were used as antioxidants instead of vitamin C (0.12%) in Tunisian beef sausages, as the samples with incorporated extracts had similar pH values compared to the control [34]. The power of the hydrogen levels increased significantly by 0.15 and 0.10, respectively, when proteins of *Spirulina* and *Chlorella* were added to replace soy in cooked turkey breasts [35] and chicken roti (minced chicken meat wrapped in whole chicken breasts and cooked in the oven) [36]. Except for the study by Marti-Quijal et al. [37], where the pH values significantly decreased, other studies [35,36,38,39,40] that replaced soy with algal proteins generally resulted in a significant increase in pH values up to 0.16 (Table 2).

### 3.2. Proximate Composition

#### 3.2.1. Moisture

Moisture has a significant impact on the quality of meat, especially on its taste, texture, and appearance, as well as on its shelf life [43]. Algae contain less moisture than meat products, and their addition can significantly reduce water content. Also, they have high dietary fiber content, which reduces moisture. In their most recent study, Bošković Cabrol et al. [25] found that by incorporating 3% white *Chlorella vulgaris* (chlorophyll-deficient microalgae mutants) into frankfurters, the moisture content decreased by nearly 1%. Furthermore, the moisture content of chicken sausages, including 6% of three different seaweeds (*Kappaphycus alvarezii*, *Sargassum polycystum*, and *Caulerpa lentilifira*), was significantly lower [44]. A similar result was observed after the incorporation of *Eucheuma cottonii* flour (7.5%) into beef meatballs when the moisture content decreased by 1% [19]. The same effect was found when Sea tangle (1%) [22] and Wakame (3%) were added to pork patties [28]. Similar to this, all other studies with low-salt meat products reported lower moisture content regardless of the amount and type of added algae [21,45,46]. In chorizo sausages, the moisture content was unaffected by the inclusion (2.6%) of the seaweed mixture (*Ulva* spp., *Gracilaria* spp., *Fucus vesiculosus*) [47]. The same was found when different algae (Sea spaghetti, Wakame, Nori, and Dulse) were added to frankfurters (1%) [48] and pork sausages (2.5%) [49]. On the other hand, the salt-reduced frankfurters, containing 1% of Sea tangle, Wakame, Hijiki (*Hizikia fusiforme*), or Glasswort (*Salicornia herbacea* L.), had significantly higher moisture content compared to the control [26] (Table 3).

Protein extracts from *Spirulina* and *Chlorella* have not resulted in any significant difference in moisture content when replacing soy protein in chicken roti [36], fresh sausages [38], turkey burgers [37], chorizo [39], and beef patties [40], while only one study reported significantly higher moisture content after the incorporation of algal protein into the brine, which was injected in turkey breasts [35] (Table 4). Similarly, when *Fucus vesiculosus* extract (0.1%) was incorporated into pork patties, no significant effect on the moisture content was observed [42]. The addition of polysaccharide extract from *Chaetomorpha linum* to Tunisian beef sausages in a concentration of 0.125% significantly increased the moisture content compared to the control with vitamin C [34].

#### 3.2.2. Fat

In the case of algae addition with higher fat levels, the products’ fat content remains the same. For example, the addition of Sea spaghetti into poultry steaks (3%) [45], pork sausages (5%) [49], and salt-reduced frankfurters [46] had no significant influence on fat levels. Also, no changes were detected in fat content when Nori was added to meat emulsion systems [21,50] and pork sausages [49]. Similarly, Wakame incorporation showed no significant difference after the addition of salt-reduced frankfurters (2%) [18] and meat emulsion systems (5%) [50]. On the other hand, the incorporation of low-fat algae decreased the lipid content in the products. White and honey *Chlorella* (3%) both showed the ability to reduce the fat content by more than 1% in frankfurters [25]. Additionally, a Sea tangle incorporation of only 1% into pork patties [22] and salt-reduced frankfurters [26] significantly decreased the fat content of the algal samples, while in breakfast sausages, it remained comparable (*p* > 0.05) [23]. *Kappaphycus alvarezii*, *Sargassum polycystum*, and *Caulerpa lentilifira* (2%) also lowered the amount of fat in chicken sausages [44]. The same was observed with *Eucheuma cottonii* incorporation (2.5%) into meatballs [19] (Table 3).

The fat content of meat products remains unaffected after the addition of algae extracts. Like the case with pork liver pâtés with added seaweed extracts (0.05%) from *Ascophyllum nodosum*, *Fucus vesiculosus*, and *Bifurcaria bifurcat* [41] and the addition of algal protein as soy replacers in fresh sausages [38], turkey burgers [37], chorizo [39], and patties [40] (Table 4).

Despite having a relatively low-fat content, integrating algae into meat products may still have positive effects on their fatty acid profile, attributed to their high content of polyunsaturated fatty acids [11]. From the reviewed literature, only one paper covered these parameters, and it was concluded that the frankfurters enriched with 3% of *Chlorella vulgaris* had higher contents of C18:2n-6, C18:3n-3, C18:3n-6 and lower amounts of C16:0 and C18:0 (*p* < 0.05) [25].

#### 3.2.3. Protein

The protein content of salt-reduced frankfurters [18] and meat emulsions [21] remained unchanged after adding Wakame in 2% and 5%, respectively. Sea spaghetti also did not affect protein content when it was added to meat emulsion systems (5%) [21], pork sausages (5%) [49], and poultry steaks (3%) [45]. The same was observed when a mixture of *Ulva* spp., *Gracilaria* spp., and *Fucus vesiculosus* was added (2.6%) to chorizo sausages [47] when Sea tangle was added to salt-reduced frankfurters (1%) [26], breakfast sausages (4%) [23], and reduced-fat pork patties (5%) [22]. In contrast, the addition of Nori (5%) increased the protein content of meat emulsion systems [21,50] and pork sausages [49]. This can be explained by the higher protein content in Nori than in other types of seaweed [21]. White and honey *Chlorella* also increased the protein content of the frankfurters [25] (Table 3).

The protein levels of liver pâtés were significantly increased after the addition of *Ascophyllum nodosum* and *Bifurcaria bifurcata* extracts at 0.05%, compared to control samples [41], while the extracts from *Fucus vesiculosus* (0.1%) had no statistically significant effect in pork patties [42]. *Spirulina* and *Chlorella* protein extracts successfully replaced soy in meat products since no significant difference in protein content was observed for reformulated cooked turkey breasts [35], chicken roti [36], fresh sausages [38], chorizo [39], and meat patties [40]. The only exceptions were turkey burgers, where a decrease was reported in products with *Chlorella* protein compared to the control [37] (Table 4).

#### 3.2.4. Ash

The ash content of meat products increased with the addition of algae due to their high mineral and vitamin matter. Therefore, the integration of honey and white *Chlorella* (3%) resulted in a significant increase in ash levels for frankfurter samples compared to the control [25]. Sea tangle in breakfast sausages (2%) [23] and salt-reduced frankfurters (1%) [26] also caused higher ash values. Similar observations were made when Wakame was incorporated in salt-reduced frankfurters (1%) [26] and beef patties (3%) [28], when *Kappaphycus alvarezii* and *Sargassum polycystum* were added to chicken sausages (6%) [44], or when *Eucheuma cottonii* flour (5%) was added to beef meatballs [19]. Contrary to this, the mixture of *Ulva* spp., *Gracilaria* spp., *Fucus vesiculosus* in chorizo sausages (2.6%) had no effect [47], while the ash levels were reduced after the addition of Sea spaghetti (2.5%) and Nori (2.5%) into the meat emulsion system [21] (Table 3).

The ash content of cooked turkey breasts [35] and beef patties [40] was significantly lower after *adding Spirulina* and *Chlorella* protein extracts instead of soy. On the other hand, when soy protein was replaced with the same extracts in fresh sausages [38], chicken roti [36], turkey burgers [37], and chorizo [39], the authors observed that there were no differences (*p* > 0.05) in samples regarding the ash content. Also, similar effects were detected when *Fucus vesiculosus* extracts (0.1%) were added to pork patties [42] (Table 4).

### 3.3. Water-Holding Capacity and Cooking Loss

Algae contain fibers with high water-holding and binding capacity, closely related to their polysaccharide composition. The type and quantity of the polysaccharides in algae’s dietary fiber fractions will determine their gelation ability [27]. Therefore, the incorporation of 2% of *Spirulina* significantly increased (54%) the water-holding capacity (WHC) of pâté samples [24]. Also, Widati et al. [19] reported that the addition of *Eucheuma cottonii* flour (5%) significantly increased the WHC in Indonesian-style beef meatballs and reduced the cooking loss (CL) compared to control batches without algae. When investigating the effect of various macroalgae at different concentrations, Mohammed et al. [49] concluded that 2.5% Sea spaghetti and Irish Wakame significantly improved the WHC of fresh pork sausages, while the CL of all samples remained unchanged. In contrast, a strong reduction in CL was observed in meat patties [22] and breakfast sausages [23] made with a 3% addition of Sea tangle. When three different types of tropical edible seaweeds (*Kappaphycus alvarezii*, *Sargassum polycystum*, and *Caulerpa lentilifira*) were added to chicken sausages, the decrease in CL was directly proportional to the concentration of algae used (2%, 4%, 6%) [44]. Another study that also used *Kappaphycus alvarezii* as an ingredient (6%) in chicken sausages made out of mechanically deboned meat reported an increase in WHC and a tenfold reduction in CL [20].

On the other hand, commercial seaweed extract, AlgySalt^®^ (used as a salt substitute), appeared to have similar binding capabilities to NaCl, which resulted in an almost identical CL in fresh pork sausages made with this extract or without it [31]. The WHC of fresh pork patties ranged from 32.3 to 37.8% and from 31.5 to 34.6% on days 2 and 7 of refrigerated storage, respectively, and were unaffected by adding laminarin and fucoidan extracts (0.01–0.5%) from *Laminaria digitata*. Cooking loss was also unaffected by the addition of the extracts [32]. When it comes to the replacement of soy protein in meat products with algal proteins, the study of Marti-Quijal et al. [38] showed that the WHC and CL in fresh pork sausages with *Spirulina* and *Chlorella* proteins were not different (*p* > 0.05) compared to control samples. A similar was observed when the same algae extracts were added to turkey burgers [37] and meat patties [40].

### 3.4. Texture

Incorporating white (3%) and honey *Chlorella* (3%) into frankfurters had no significant effect on hardness and chewiness at the beginning of cold storage, while they increased by the end of the 60th day. However, only samples with white *Chlorella* showed a significant decrease in cohesiveness and springiness [25]. The addition of Sea tangle (3%) into breakfast sausages increased the hardness and chewiness [23], while in pork patties (3%), the addition of this seaweed showed a significant increase in hardness, springiness, and chewiness [22]. Compared to the control samples, mechanically deboned chicken meat sausages made with 2% *Kappaphycus alvarezii* were harder and chewer [20]. Pork sausages with Sea spaghetti showed a significant increase in hardness and chewiness when this seaweed was added (5%), while it took only 1% of Nori to achieve the same effect [49]. The incorporation of Nori (2.5%) into meat batters also increased the hardness and chewiness, while the same concentration of Wakame resulted in even greater hardness and chewiness [21]. The variety and quantity of dietary fibers in these algae may cause the changed texture profile parameters.

However, since algae extracts have different types and lower levels of soluble fibers compared to whole algae, a distinct influence on the texture of meat products was observed. For example, when *Spirulina* and *Chlorella* protein extracts were added as soy protein substitutes in cooked turkey breasts [35], chicken roti [36], and fresh sausages [38], modified products had significantly lower hardness, springiness, cohesiveness, and chewiness. On the other hand, the inclusion of *Palmaria palmata* extract into the brine did not have any effects (*p* > 0.05) on the texture of salt-reduced cooked hams [51]. Likewise, laminarin and fucoidan extract from *Laminaria digitata* did not change (*p* > 0.05) the textural parameters of fresh pork patties, although their hardness started decreasing after the second day of cold storage [32].

### 3.5. Color

Having a high pigment content, micro- and macroalgae both influence the color of the products they are added to. Depending on the dominant chlorophylls, carotenoids, and phycocyanin present, the algae can be green, blue-green, red, brown, or golden brown [11,12]. These pigments are used as nutraceutical ingredients and food colorants because they possess important qualities as biologically active agents [52]. This is why the inclusion of white and honey *Chlorella* (3%) into frankfurters led to a decrease in redness and an increase in yellowness, as can be seen in Table 5, while the lightness was significantly lower only in samples with honey *Chlorella* during the time of refrigerated storage [25]. Also, significant color changes were observed when Sea tangle was added to reduced-fat pork patties [22] and breakfast sausages [23]. Uncooked and cooked reduced-fat pork patties’ lightness and redness values were significantly lower in samples containing Sea tangle (1%) compared to the control, and these parameters decreased even more with increasing algal content. The opposite was observed regarding the yellowness of the samples [22]. Identical trends in CIEL*a*b* color changes were reported when Sea tangle (1%) was incorporated into breakfast sausages [23].

According to Cofrades et al. [21], the lightness and redness were reduced, and the yellowness increased significantly with the addition of Sea spaghetti (2.5%) to the cooked pork gel/emulsion system. The same algae in the same concentration were added to fresh pork sausages, and the lightness and yellowness were not significantly affected, while the redness decreased significantly [49]. When a lower concentration (1%) of Sea spaghetti was added to reformulated frankfurters, the total color difference (Δ*E*) higher than 2 was observed. The main cause for such total color differences was significant changes in redness, while yellowness and lightness remained unaffected by the addition of algae [48]. However, the same concentration (1%) of Wakame added to the same product resulted in a much higher Δ*E* of 5.3 because all three color components (L*, a*, b*) significantly decreased [48]. Surprisingly, when Wakame (2.5%) was used as an ingredient in a cooked pork gel/emulsion system, lightness and redness also decreased, but yellowness was significantly higher [21]. The addition of Nori significantly reduced all three color parameters when it was added to reformulated frankfurters at 1% [48], at 2.5% into fresh sausages [49], and at 5% into cooked pork gel/emulsion system [21]. The same was reported for fresh sausages [49] and reformulated frankfurters [48] with 2.5% Dulse.

**Table 5:** Differences in color parameters after whole algae incorporation.

Meat Product	Algae Type	Inclusion Level	ΔL*	Δa*	Δb*	Reference
Frankfurters	White *Chlorella*	3%	↓	↓	↑	Bošković Cabrol et al., 2023 [25]
	Honey *Chlorella*	3%	↓	↓	↑	
Frankfurters	Wakame	2%	↓	↓	↑	Choi et al., 2017 [18]
Pork patties	Sea tangle	5%	↓	↓	↑	Choi et al., 2012 [22]
Frankfurters	Sea tangle	1%	↓	↓	↑	Choi et al., 2015 [26]
	Wakame	1%	↓	↓	↑	
	Hijiki	1%	↓	↓	↑	
	Glasswort	1%	↓	↑	↑	
Pork gel/emulsion system	Sea spaghetti	2.5%	↓	↓	↑	Cofrades et al., 2008 [21]
	Wakame	2.5%	↓	↓	↑	
	Nori	2.5%	↓	↓	↑	
Poultry steaks	Sea spaghetti	3%	↓	↓	↑	Cofrades et al., 2011 [45]
Beef patties	Sea spaghetti	10%	↑	↓	↑	Cox S. and Abu-Ghannam N., 2013 [27]
Breakfast sausages	Sea tangle	4%	↓	↓	↑	Han et al., 2010 [23]
Pork sausages	Sea spaghetti	2.5%	↓	↓	↓	Mohammed et al., 2022 [49]
	Irish wakame	2.5%	↓	↓	↓	
	Dulse	2.5%	↓	↓	↓	
	Nori	2.5%	↓	↓	↓	
Chicken sausage	*K. alvarezii*	6%	↓	↑	↓	Munsu et al., 2021 [44]
	*S. polycystum*	6%	↓	↑	↓	
	*C. lentilifira*	6%	↓	↓	↓	
MDCM chicken sausages	*K. alvarezii*	6%	↓	↑	NSD	Pindi et al., 2017 [20]
Frankfurters	Sea spaghetti	1%	↓	↓	↑	Vilar et al., 2020 [48]
	Wakame	1%	↓	↓	↓	
	Nori	1%	↓	↓	↓	
	Dulse	1%	↓	↓	↓	
Beef meatballs	*E. cottonii*	7.5%	↓	↓	↓	Widati et al., 2021 [19]

↑—the addition of whole algae increased the value of the parameter; ↓—the addition of whole algae decreased the value of the parameter; NSD—no significant difference.

Incorporating *Spirulina* and *Chlorella* protein extracts (1%) instead of soy in cooked turkey breasts resulted in lower L* and a* values, while b* values became higher compared to control samples [35]. Conversely, b* values were lower when the same microalgae extracts were added (1%) to chicken roti for the same purpose [36]. A significant decrease in all three color parameters was observed when these extracts were added to fresh pork sausages (1%) [38], turkey burgers (1%) [37], chorizo sausages (3%) [39], and beef patties (1%) [40] (Table 6). The addition of *Spirulina* extract (5%) to pork sausages significantly decreased the lightness and yellowness, both on day 0 and day 18 of refrigerated storage. Adding the extract did not change the initial redness, but it was significantly higher by the end of the refrigerated storage time [30].

After the incorporation of *Fucus vesiculosus* extracts as natural antioxidants in pork patties, the lightness, redness, and yellowness did not change (*p* > 0.05), even with 250 mg/kg of the added extract, and over the storage time of 18 days [42]. These findings highlight the potential of *Fucus vesiculosus* extracts to prevent color loss during the refrigerated storage of pork patties. Similarly, the redness of the turkey sausages was maintained during 15 days of refrigeration with the addition of 0.04% *Cystoseira barbata* extract. Compared to the control, this change to the product composition increased the yellowness and decreased the lightness of the sausages, which was related to the extract’s yellowish color [53].

**Table 6:** Differences in color parameters after algal extracts incorporation.

Meat Product	Algae Type	Inclusion Level	ΔL*	Δa*	Δb*	Reference
Chinese sausage	*Spirulina* PS	0.5%	↓	↑		Luo et al., 2017 [29]
Chinese sausage	*Spirulina* ex.	5%	↓	NSD	↓	Luo et al., 2017 [30]
Fresh sausage	*Spirulina* protein	1%	↓	↓	↓	Marti-Quijal et al., 2019 [38]
	*Chlorella* protein	1%	↓	↓	↓	
Turkey burger	*Spirulina* protein	1%	↓	↓	↓	Marti-Quijal et al., 2019 [37]
	*Chlorella* protein	1%	↓	↑	NSD	
Cooked turkey breast	*Spirulina* protein	1%	↓	↓	↑	Marti-Quijal et al., 2018 [35]
	*Chlorella* protein	1%	↓	↓	↑	
Ground pork meat	*H. pluvialis* ex.	0.05%	↓	↑	↑	Pogorzelska et al., 2018 [54]
Chicken roti	*Spirulina* protein	1%	↓	↓	↓	Parniakov et al., 2018 [36]
	*Chlorella* protein	1%	↓	↓	↓	
Chorizo sausages	*Spirulina* protein	3%	↓	↓	↓	Thirumdas et al., 2018 [39]
	*Chlorella* protein	3%	↓	↓	↓	
Beef patties	*Spirulina* protein	1%	↓	↓	↓	Zugcic et al., 2018 [40]
	*Chlorella* protein	1%	↓	↓	↓	
Pork liver pâté	*A. nodosum* ex.	0.05%	NSD	NSD	NSD	Agregan et al., 2018 [41]
	*F. vesiculosus* ex.		NSD	NSD	NSD	
	*B. bifurcata* ex.		NSD	NSD	NSD	
Pork patties	*F. vesiculosus* ex.	0.1%	↓	↓	↓	Agregan et al., 2019 [42]
Beef sausages	*Ch. linum* PS	0.25%	↓	↑	↓	Hamzaoui et al., 2020 [34]
Pork patties	*Ulva lactuca* and *Ulva rigida* ex.	0.1%	↓	↓	↓	Lorenzo et al., 2014 [33]
Pork patties	*L. digitata* ex.	0.5%	↓	↓	↑	Moroney et al., 2013 [32]
MDCM turkey sausages	*C. barbata* ex.	0.04%	↓	↑	↑	Sellimi et al., 2017 [55]
MDCM turkey sausages	*C. barbata* ex.	0.04%	↓	↑	↑	Sellimi et al., 2018 [53]
Turkey sausages	AlgySalt^®^	2%	NSD	NSD	NSD	Triki et al., 2017 [31]

↑—the addition of extracts increased the value of the parameter; ↓—the addition of extracts decreased the value of the parameter; NSD—no significant difference.

### 3.6. Sensory Evaluation

The inclusion of algae, as a non-traditional ingredient with strong and specific flavor, may adversely affect the sensory attributes of reformulated products, as the traditional formulation’s ingredients play a crucial role in shaping the typical sensory characteristics preferred by consumers for each product. However, there were no significant differences in flavor and the juiciness of fresh pork sausages made with 3% Sea tangle, while the springiness, hardness, and overall sensory acceptability were improved by the addition of this macroalga [56]. Contrarily, the same seaweed in the same concentration, but this time used as an ingredient in fat-reduced pork patties [22] or in breakfast sausages [23], exhibited no significant difference in overall sensory acceptability compared to the control samples. Finally, Han et al. [23] reported that the sensation of breakfast sausages with 1% Sea tangle was accepted by the consumers. The addition of Wakame (2%) in frankfurters resulted in significantly lower scores for the flavor, juiciness, and tenderness of frankfurters [18]. The scores for flavor were also reduced when *Kappaphycus alvarezii*, *Sargassum polycystum*, and *Caulerpa lentilifira* were added (2%, 4%, 6%) to chicken sausages [44]. Overall sensory acceptability significantly decreased in cooked sausages containing Dulse at 2.5% and 5% and Sea spaghetti at 5% [49], as well as frankfurters containing 1% of Wakame, Nori, and Dulse [48]. To prevent these adverse effects of algae on the sensory acceptability of meat products, Voloschenko et al. [24] recommended the addition of ≤2% of *Spirulina* in pâtés. Color scores were significantly decreased after the incorporation of Sea tangle (3%) in reduced-fat pork patties [22] and breakfast sausages [23] and after the addition of Wakame (2%) in frankfurters. Chicken sausages with *Sargassum polycystum* and *Caulerpa lentilifira* had lower color values in direct proportion to the added amount of algae (2–6%), while samples with *Kappaphycus alvarezii* had no significant color difference regardless of the amount of algae added [44].

The extract of polysaccharides from *Spirulina* improved flavor and overall sensory acceptability (*p* < 0.05) when added in quantities up to 0.5% into Chinese-style sausages, as a result of polysaccharides ability to protect the products from lipid peroxidation [29]. Similarly, the sensory attributes of pork sausages, including color, flavor, and overall acceptability, were significantly improved during storage time, even with the highest concentration (5%) of *Spirulina* extract. The TBARS values (Thiobarbituric Acid Reactive Substances) appeared to correlate with these sensory results [30]. *Chlorella* and *Spirulina* could be advantageous candidates for producing new meat products, as the flavor profile of the beef patties was not significantly affected by substituting soy protein with algal proteins [40]. However, chicken roti made with *Spirulina* and *Chlorella* proteins had lower acceptability scores (*p* < 0.05) compared to products with soy protein [36]. The addition of astaxanthin extract from *Haematococcus pluvialis* greatly influenced ground pork meat’s sensory acceptability. The meat with the highest extract concentrations (0.45 g/kg) had the best rating for overall color acceptability [54]. Pork patties with 0.01% laminarin and fucoidan extract from *Laminaria digitata* had acceptable sensory scores [32]. On the other hand, pork patties with *Fucus vesiculosus* extract (0.1%) showed no significant difference in color and odor over 18 days of storage [42].

### 3.7. Microbiology

White and honey *Chlorella*-enriched (3%) frankfurters showed significantly lower total viable counts (TVC) and psychotropic bacteria and lactic acid bacteria (LAB) counts at the end of the refrigerated storage (60 days), indicating an antimicrobial effect of these microalgae [25]. Similarly, pork sausages with Sea tangle (3%) had nearly 20% lower numbers of total aerobic bacteria compared to control samples by the 14th day of the cold storage period [56]. Also, Sea spaghetti showed a positive influence on the microbiology of cooked beef patties when added in an unusually high concentration (>10%), resulting in significantly lower TVC by the end of cold storage [27]. The lower bacterial count may be attributed to the presence of phenolic compounds, widely recognized as antibacterial substances in algae. On the other hand, the addition of Wakame (3%) into beef patties did not result in changes (*p* > 0.05) in levels of TVC and *Enterobacteriaceae* after 152 days of frozen storage [28]. Only one study reported the unwanted effect of added algae on the microbiological quality of meat products, where significantly higher values were found for TVC and LAB counts and *Enterobacteriaceae* in low-salt poultry steaks after the addition of Sea spaghetti (3%) [45]. These results may be explained by the fact that the poultry steaks with algae had three times lower salt content than the control samples.

Polysaccharide extract from *Chaetomorpha linum* had a better antimicrobial effect than 0.125% of vitamin C in Tunisian sausages since the same concentration of the extract showed significantly lower *Salmonella* and *Listeria* counts at the end of cold storage (12 days), while the counts of total coliforms and mesophilic flora were lower [34]. The addition of *Ascophyllum nodosum*, *Fucus vesiculosus*, and *Bifurcaria bifurcate* extract (0.05%) to low-fat pork liver pâtés had no effect (*p* > 0.05) on TVC after 180 days of refrigerated storage compared to control sample with 50 mg/kg butylated hydroxytoluene (BHT) [41]. Likewise, laminarin and fucoidan extracts from *Laminaria digitata* were without antibacterial activity in fresh pork patties since the TVCs were similar for all samples (*p* > 0.05) [32]. Other results showed that AlgySalt^®^, a natural salt substitute developed from seaweed extracts for sodium reduction, has a preservative effect similar to NaCl when added to cooked sausages [31]. Similarly, adding *Spirulina* polysaccharide extracts (0.5%) to Chinese-style sausages had no influence on microbial growth during the product’s shelf life [29].

### 3.8. Antioxidant Activity

Meat is highly sensitive to oxidation processes, which can result in a loss of quality during food storage and distribution [34]. To prevent this, some natural antioxidants can be added to the formulation of the final product, including whole algae or their extracts. This is why *Spirulina*-enriched (1%) pork liver pâtés had significantly lower peroxide numbers compared to the control, directly proportional to the concentration of algae added [24]. In frankfurters with white and honey *Chlorella* (3%), although peroxide values were significantly higher during the storage period, probably due to the higher content of polyunsaturated fatty acids (PUFA) originating from algae, significantly lower TBARS values were observed in almost all stages of the refrigerated storage [25]. The addition of Sea tangle (3%) into pork sausages decreased TBARS values after 14 days of storage [56]. Similarly, chicken sausages with 2% added of *Kappaphycus alvarezii*, *Sargassum polycystum*, and *Caulerpa lentilifira* had significantly lower TBARS values at the end of the storage period (28 days) [44], while in mechanically deboned chicken meat sausages, *Kappaphycus alvarezii* showed a similar impact at the same concentration [20]. Only one study found a negative effect of algae addition on lipid oxidation in meat products, where frankfurters made with 1% Sea spaghetti had higher TBARS levels, while the other three seaweed formulations (1% Nori, Dulse, and Wakame) showed no significant difference compared to control samples after 63 days of cold storage [48].

On the other hand, cooked pork patties containing laminarin and fucoidan extract (0.5%) from *Laminaria digitata* exhibited lower lipid oxidation after 14 days of chilled storage compared to samples without extract [32]. Similarly, the addition of *Cystoseira barbata* extract (0.02%) extended the shelf life of the mechanically separated turkey meat sausages because of reduced TBARS values during the refrigerated storage (15 days) [53]. Also, the incorporation of *Ascophyllum nodosum*, *Fucus vesiculosus*, and *Bifurcaria bifurcate* extract (0.05%) into low-fat pork liver pâtés resulted in a significant decrease in TBARS at the end of the storage period (180 days) [41]. Chinese-style sausages with polysaccharide extracts (0.5%) from *Spirulina* also lowered lipid oxidation levels by the end of the chilled storage (24 days) compared to control samples [29], while pork patties with 0.1% *Fucus vesiculosus* extract showed higher oxidative stability at the end of the storage period (18 days) [42].

## 4. Conclusions

In most of the studies, algae mainly increased the pH values of the meat products by approximately 0.1 and up to 0.3 during refrigerated storage. These pH changes are relatively small and may have moderately contributed to changes in WHC and CL. Protein content was not significantly altered with the addition of algae or their extracts, in general. Due to algae’s relatively low-fat content (5% of dry weight on average), the total lipids are either slightly reduced or unchanged when added ’on top’ of meat products. Adding algae to meat products resulted in lower moisture and higher ash content due to their high dietary fiber content. The examples with lower ash content in the samples (meat emulsion systems and frankfurters) after the addition of algae were left without a plausible explanation. The extracts had no influence on both moisture and ash content. Most studies discovered that adding seaweed to the products significantly increased their hardness and chewiness, while springiness and cohesiveness were unaffected or minimally changed in most cases. Whole algae and their extracts reduced the lightness and redness of meat products, while changes in yellowness depended on the type of algae.

Different algal sources affected the sensory properties, significantly impacting the panelists’ acceptance. In general, the overall acceptability of meat products was lower after the inclusion of algae. However, samples with Sea tangle revealed acceptable or even slightly higher scores at concentrations between 1 and 3%. The extracts showed a milder effect on the sensory characteristics, including samples with positive changes after different polysaccharide extract implementation.

Based on the studies found, it can be concluded that some algae species had a positive effect on the microbiology of meat products during refrigerated storage, while up to 0.1% of polysaccharide extracts from algae can achieve the same or even better effect as more commonly used traditional preservatives. Several studies determined that up to 3% of micro- and macroalgae have the potential to decrease lipid oxidation in meat products. At the same time, algal extracts showed strong antioxidant activity during cold storage in general.

The acceptance of this food faces obstacles among consumers, primarily attributed to its distinctive qualities, especially its sensory characteristics, and limited consumer awareness. It is crucial to comprehend consumer perceptions of such food and identify the factors impacting the purchasing decisions for meat products based on micro- and macroalgae. However, this type of knowledge is largely missing from substantial parts of the world (like Eastern Europe, South America, or Africa) and available almost exclusively to Western countries.

Viewed through the lens of sustainable resource development, incorporating algae into meat and other food industries has the potential to enhance the creation of high-nutrient products. However, it is essential to acknowledge potential drawbacks. Utilizing algae on a large scale as a dietary supplement requires careful consideration of the potential species invasion and the ecological impact stemming from its unchecked growth.

Simultaneously, it is crucial to investigate the process of nutrient digestion and absorption from algae within the human body to comprehend its bioavailability. Additionally, given the diverse range of algae species, it is imperative to conduct both in vivo and in vitro experiments to gather information regarding the toxicity and allergic reactions associated with different algae species.

## Figures and Tables

**Table 1:** Differences in pH after whole algae incorporation.

Meat Product	Algae Type	Inclusion Level	Difference in pH	Reference
Frankfurters	White *Chlorella*	3%	↑	Bošković Cabrol et al., 2023 [25]
	Honey *Chlorella*	3%	↑	
Pork liver pâté	*Spirulina*	2.5%	NSD	Voloschenko et al., 2021 [24]
Frankfurters	Wakame	2%	↑	Choi et al., 2017 [18]
Pork patties	Sea tangle	5%	↓	Choi et al., 2012 [22]
Frankfurters	Sea tangle	1%	↑	Choi et al., 2015 [26]
	Wakame	1%	↑	
	Hijiki	1%	↑	
	Glasswort	1%	↑	
Pork gel/emulsion system	Sea spaghetti	2.5%	NSD	Cofrades et al., 2008 [21]
	Wakame	2.5%	↑	
	Nori	2.5%	↓	
Beef patties	Sea spaghetti	10%	NSD	Cox S. and Abu-Ghannam N., 2013 [27]
Breakfast sausages	Sea tangle	4%	↓	Han et al., 2010 [23]
Beef patties	Wakame	3%	↑	López-López et al., 2010 [28]
MDCM chicken sausages	*K. alvarezii*	6%	↑	Pindi et al., 2017 [20]
Beef meatballs	*E. cottonii*	7.5%	↑	Widati et al., 2021 [19]

↑—the addition of whole algae increased the value of the parameter; ↓—the addition of whole algae decreased the value of the parameter; NSD—no significant difference.

**Table 2:** Differences in pH after algal extracts incorporation.

Meat Product	Algae Type	Inclusion Level	Difference in pH	Reference
Chinese sausage	*Spirulina* PS	0.5%	NSD	Luo et al., 2017 [29]
Chinese sausage	*Spirulina* ex.	5%	NSD	Luo et al., 2017 [30]
Fresh sausage	*Spirulina* protein	1%	↓	Marti-Quijal et al., 2019 [38]
	*Chlorella* protein	1%	↑	
Turkey burger	*Spirulina* protein	1%	↓	Marti-Quijal et al., 2019 [37]
	*Chlorella* protein	1%	↓	
Cooked turkey breast	*Spirulina* protein	1%	↑	Marti-Quijal et al., 2018 [35]
	*Chlorella* protein	1%	↑	
Chicken roti	*Spirulina* protein	1%	↑	Parniakov et al., 2018 [36]
	*Chlorella* protein	1%	↑	
Chorizo sausages	*Spirulina* protein	3%	↑	Thirumdas et al., 2018 [39]
	*Chlorella* protein	3%	↑	
Beef patties	*Spirulina* protein	1%	NSD	Zugcic et al., 2018 [40]
	*Chlorella* protein	1%	↑	
Pork liver pâté	*A. nodosum* ex.	0.05%	NSD	Agregan et al., 2018 [41]
	*F. vesiculosus* ex.		NSD	
	*B. bifurcata* ex.		NSD	
Pork patties	*F. vesiculosus* ex.	0.1%	↑	Agregan et al., 2019 [42]
Beef sausages	*Ch. linum* PS	0.25%	↑	Hamzaoui et al., 2020 [34]
Pork patties	*Ulva lactuca* and *Ulva rigida* ex.	0.1%	↓	Lorenzo et al., 2014 [33]
Pork patties	*L. digitata* ex.	0.5%	NSD	Moroney et al., 2013 [32]
Turkey sausages	AlgySalt^®^	2%	NSD	Triki et al., 2017 [31]

↑—the addition of extracts increased the value of the parameter; ↓—the addition of extracts decreased the value of the parameter; NSD—no significant difference.

**Table 3:** Differences in proximate composition after whole algae incorporation.

Meat Product	Algae Type	Inclusion Level	Moisture	Ash	Protein	Fat	Reference
Frankfurters	White *Chlorella*	3%	↓	↑	↑	↓	Bošković Cabrol et al., 2023 [25]
	Honey *Chlorella*	3%	NSD	↑	↑	↓	
Pork liver pâté	*Spirulina*	2.5%	↑				Voloschenko et al., 2021 [24]
Frankfurters	Wakame	2%	NSD	↑	NSD	NSD	Choi et al., 2017 [18]
Pork patties	Sea tangle	1%	↓	↑	NSD	↓	Choi et al., 2012 [22]
Frankfurters	Sea tangle	1%	↑	↑	NSD	↓	Choi et al., 2015 [26]
	Wakame	1%	↑	↑	NSD	↓	
	Hijiki	1%	↑	↑	NSD	↓	
	Glasswort	1%	↑	↑	NSD	↓	
Pork gel/emulsion system	Sea spaghetti	2.5%	NSD	↓	NSD	NSD	Cofrades et al., 2008 [21]
	Wakame	2.5%	↑	↓	NSD	NSD	
	Nori	2.5%	↓	↓	↑	NSD	
Poultry steaks	Sea spaghetti	3%	NSD	↓	NSD	NSD	Cofrades et al., 2011 [45]
Breakfast sausages	Sea tangle	2%	NSD	↑	NSD	NSD	Han et al., 2010 [23]
Meat emulsion system	Nori	5.6%	↓	↓	↑	NSD	López-López et al., 2009 [50]
	Wakame	5.6%	↓	NSD	NSD	NSD	
	Sea spaghetti	5.6%	↓	NSD	NSD	NSD	
Frankfurters	Sea spaghetti	5.5%	↓	↑	NSD	NSD	López-López et al., 2009 [46]
Beef patties	Wakame	3%	↓	↑	NSD	↓	López-López et al., 2010 [28]
Chorizo	*Ulva* spp.	2.6%	NSD	NSD	NSD	NSD	Marcal et al., 2021 [47]
	*Gracilaria* spp.						
	*F. vesiculosus*						
Pork sausages	Sea spaghetti	2.5%	NSD	↑	NSD	NSD	Mohammed et al., 2022 [49]
	Irish wakame	2.5%	NSD	↑	NSD	NSD	
	Dulse	2.5%	NSD	↑	NSD	NSD	
	Nori	2.5%	NSD	NSD	NSD	NSD	
Chicken sausage	*K. alvarezii*	6%	↓	↑	NSD	NSD	Munsu et al., 2021 [44]
	*S. polycystum*	6%	↓	↑	NSD	NSD	
	*C. lentilifira*	6%	↓	NSD	NSD	NSD	
Frankfurters	Sea spaghetti	1%	NSD	↓	NSD	↓	Vilar et al., 2020 [48]
	Wakame	1%	NSD	NSD	NSD	↓	
	Nori	1%	NSD	↓	NSD	↓	
	Dulse	1%	NSD	↓	NSD	↓	
Beef meatballs	*E. cottonii*	5%	↓	↑	↓	↓	Widati et al., 2021 [19]

↑—the addition of whole algae increased the value of the parameter; ↓—the addition of whole algae decreased the value of the parameter; NSD—no significant difference.

**Table 4:** Differences in proximate composition after algal extracts incorporation.

Meat Product	Algae Type	Inclusion Level	Moisture	Ash	Protein	Fat	Reference
Fresh pork sausage	*Spirulina* protein ex.	1%	NSD	NSD	NSD	NSD	Marti-Quijal et al., 2019 [38]
	*Chlorella* protein ex.	1%	NSD	NSD	NSD	NSD	
Turkey burger	*Spirulina* protein ex.	1%	NSD	NSD	↓	NSD	Marti-Quijal et al., 2019 [37]
	*Chlorella* protein ex.	1%	NSD	NSD	↓	NSD	
Cooked turkey breast	*Spirulina* protein ex.	1%	↑	↓	NSD	NSD	Marti-Quijal et al., 2018 [35]
	*Chlorella* protein ex.	1%	↑	↓	NSD	NSD	
Chicken roti	*Spirulina* protein ex.	1%	NSD	NSD	NSD	↑	Parniakov et al., 2018 [36]
	*Chlorella* protein ex.	1%	NSD	NSD	NSD	NSD	
Chorizo sausages	*Spirulina* protein ex.	3%	NSD	NSD	NSD	NSD	Thirumdas et al., 2018 [39]
	*Chlorella* protein ex.	3%	NSD	NSD	NSD	NSD	
Beef patties	*Spirulina* protein ex.	1%	NSD	↓	NSD	NSD	Zugcic et al., 2018 [40]
	*Chlorella* protein ex.	1%	NSD	↓	NSD	NSD	
Pork liver pâté	*A. nodosum* ex.	0.05%	NSD		↑	NSD	Agregan et al., 2018 [41]
	*F. vesiculosus* ex.	0.05%	NSD		NSD	NSD	
	*B. bifurcata* ex.	0.05%	NSD		↑	NSD	
Pork patties	*F. vesiculosus* ex.	0.1%	NSD	NSD	NSD	NSD	Agregan et al., 2019 [42]
Beef sausages	*Ch. linum* PS	0.12%	↑				Hamzaoui et al., 2020 [34]

**↑**—the addition of extracts increased the value of the parameter; **↓**—the addition of extracts decreased the value of the parameter; NSD—no significant difference.

## Data Availability

The original contributions presented in the study are included in the article, further inquiries can be directed to the corresponding authors.
